# Combining NMR and small angle X-ray scattering for the study of biomolecular structure and dynamics

**DOI:** 10.1016/j.abb.2017.05.005

**Published:** 2017-08-15

**Authors:** Haydyn D.T. Mertens, Dmitri I. Svergun

**Affiliations:** EMBL Hamburg c/o DESY, Building 25A, Notkestr. 85, Hamburg 22603, Germany

**Keywords:** SAXS, NMR, Proteins, Structure, Dynamics

## Abstract

Small-angle X-ray scattering (SAXS) and Nuclear Magnetic Resonance (NMR) are established methods to analyze the structure and structural transitions of biological macromolecules in solution. Both methods are directly applicable to near-native macromolecular solutions and allow one to study structural responses to physical and chemical changes or ligand additions. Whereas SAXS is applied to elucidate overall structure, interactions and flexibility over a wide range of particle sizes, NMR yields atomic resolution detail for moderately sized macromolecules. NMR is arguably the most powerful technique for the experimental analysis of dynamics. The joint application of these two highly complementary techniques provides an extremely useful approach that facilitates comprehensive characterization of biomacromolecular solutions.

## Highlights

• SAXS and NMR are effective and highly complementary techniques in structural biology.
• Constraints from SAXS can be readily incorporated in NMR structure calculations.
• High resolution NMR models of domains can serve as building blocks for SAXS-based rigid body modeling.
• Flexible systems can be well described using ensemble approaches combining SAXS and NMR.
• Dynamics studies can be enhanced by combining SAXS and NMR.

## 1 Introduction

Hybrid approaches in structural biology combining complementary techniques can generate results that lead to the development of much greater insight than if the individual techniques are applied alone. The combination of high-resolution structural methods such as NMR, providing atomic scale structural and dynamics information, with lower resolution methods such as SAXS that yield nanometer scale structure, is a powerful approach for the study of biomacromolecules in near native environments. In particular, NMR and SAXS are considered the most appropriate tools for the study of highly dynamic and flexible systems, which for both modern electron microscopy (EM) and X-ray crystallography remain problematic. While NMR structure determination is limited in applicability to moderately sized macromolecules (eg. < 50 kDa), the lack of size limitation for SAXS facilitates application of a hybrid structural approach of both NMR and scattering to an almost unlimited range of complex systems.

**Fig. 1:**
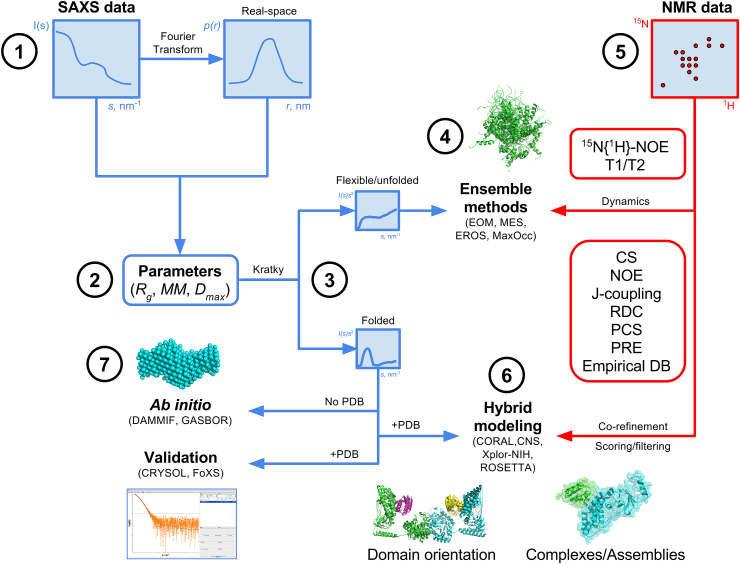
*Summary of complementarity of NMR and SAXS for structure determination/characterization*. SAXS data is recorded and real-space representations obtained via indirect Fourier transform methods **(1)**, providing qualitative information on particle shape. SAXS parameters are determined directly from the reciprocal space and real space data providing information on sample homogeneity and particle size and shape **(2)**. Kratky plot representation of the SAXS data facilitates a qualitative estimate of foldedness and/or flexibility, suggesting the appropriate path for further modeling and structural analysis **(3)**. SAXS data can be used for the analysis of dynamic systems via ensemble approaches **(4)** and is particularly powerful when combined with NMR data **(5)**, or directly incorporated in co-refinement algorithms (eg. hybrid modeling) and model scoring/filtering methods (eg. docking) **(6)**. Several modern programs used for analysis are indicated in parentheses.

There are several ways to combine NMR and SAXS data for hybrid modeling (Fig. 1). First, SAXS can be used for model validation where structures solved by high-resolution methods (e*g.* NMR, cryo-EM or X-ray crystallography) are checked for agreement with the solution scattering data. Further, the positions and orientations of protein domains or stretches of nucleic acids in complexes can be refined using SAXS-driven rigid body modeling routines. More sophisticated approaches introduce the scattering data into structure calculation routines as an additional penalty term, alongside penalties for the satisfaction of distance and orientation constraints from solution NMR such as NOEs and RDCs, respectively. In this review we will focus on SAXS, however, the methods and approaches outlined herein are readily applied to the small-angle scattering of neutrons (SANS), where additional information can be gained by H/D exchange [1].

Aside from limitations on the total size of the biomacromolecule to be studied by NMR, arising primarily from increased complexity in the recorded spectra and signal broadening, long-range distance information (>5A) is often challenging to obtain. While orientational information between domains can be obtained from measurements in alignment media (yielding residual dipolar couplings, RDCs), without long range distance information, derived from paramagnetic labels for example, it can be difficult to place these domains relative to each other. The SAXS-generated size and shape information helps to overcome this problem by imposing additional constraints. In this way the complementarity of orientational and short-range distance data from NMR and the long-range distance and shape information from SAXS can be fully exploited.

## 2 SAXS basics and parameters

**Fig. 2:**
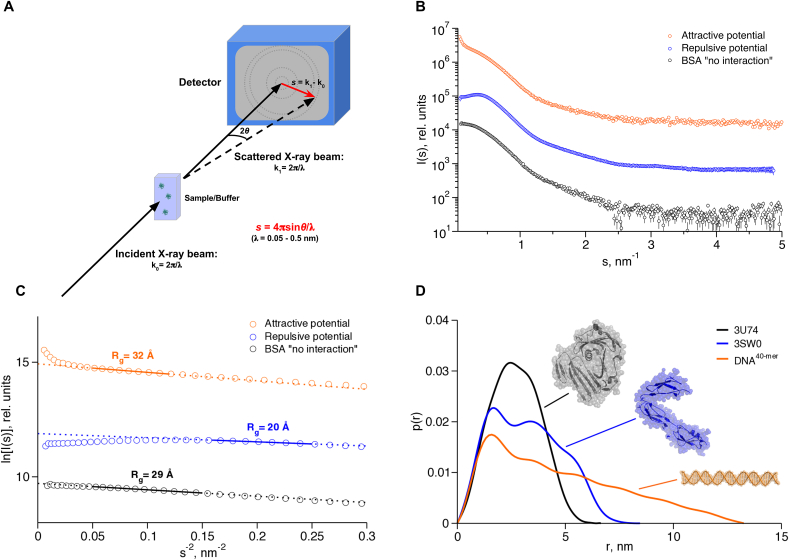
*The SAXS experiment*. Solutions of biological macromolecules are exposed to a collimated beam of X-rays, and the scattered secondary waves produced after interaction with the sample are registered on a position-sensitive detector as the momentum transfer *s* = 4πsinθ/λ **(A)**. Concentration dependent effects as observed for BSA (black circles: ideal sample, blue circles: repulsive interactions, orange circles: attractive interactions/aggregation) in the SAXS profile (**B**) and Guinier plot **(C)**. Real-space distance distributions *p(r)*, for a compact (black line and gray model), multidomain (blue) and extended (orange) biomolecule **(D)**. (For interpretation of the references to colour in this figure legend, the reader is referred to the web version of this article.)

The SAXS experiment is deceptively simple and involves exposure of a sample to a collimated beam of incident radiation of a defined sub-nm wavelength (typically 0.05–0.5 nm) (Fig. 2A). At small-angles (0.1–5°) the dominant scattering process is elastic, where no net energy transfer between the incident and scattered waves is observed. Other scattering phenomena, *eg.* Compton and Raman scattering of X-rays contribute in the most part to the background of the experiment and will not be discussed here.

Scattering of X-rays by a solution of biomolecules is dependent on the number of particles in the illuminated volume (*ie.* to the solute concentration), the square of the volume of the individual particle (*ie.* to particle size) and the square of the excess scattering length density or *contrast*. The excess scattering length density, Δ*ρ(r)* relates to the difference in the electron density of the solute and solvent. For proteins, the former value is about 410 e/nm^3^ and only slightly exceeds that of water (334 e/nm^3^). That biomolecules are weak scatterers with low contrast means that synchrotron SAXS beamlines and laboratory sources must be optimized for the minimization of the contribution of background. Rather stringent requirements are also imposed on the sample and its preparation. So, while theoretically straightforward, the SAXS experiment as applied to biological systems is a challenging task.

Dilute aqueous solutions of proteins, nucleic acids or other macromolecules give rise to an isotropic scattering intensity, which depends on the modulus of the momentum transfer ***s*** (*s* = 4πsin(θ)/λ, where *2θ* is the angle between the incident and scattered beam):

(1) $$I\left(s\right)={\langle \mathrm{I(s)}\rangle }_{\Omega }={\langle \mathrm{A(s)A}*\mathrm{(s)}\rangle }_{\Omega }$$

where the scattering amplitude **A**(s) is a Fourier transformation of the excess scattering length density of the particle Δ*ρ*(***r***), and the scattering intensity is averaged over all orientations (Ω). The background corrected intensity *I*(*s*) is proportional to the scattering of a single particle averaged over all orientations (see books and reviews [1], [2], [3], [4] for more details).

The scattered X-rays are usually registered on two-dimensional area detectors, and radially averaged to 1D SAXS profiles for analysis (Fig. 2B). Difference profiles obtained after subtraction of solvent scattering are used for the direct extraction of important parameters, providing information about the size, oligomeric state and overall shape of the molecules in solution. These parameters include: the molecular mass (*MM*), radius of gyration (*R*_*g*_), hydrated particle volume (*V*_*p*_) and maximum particle diameter (*D*_max_). The *R*_*g*_ is the average of the squared center-of-mass distances in a particle weighted by the electron density and provides an indication of particle compactness. For a monodisperse solution at the smallest angles the Guinier equation (Guinier, 1939) holds:

(2) $$I\left(s\right)=I\left(0\right)\exp\left(-\frac{1}{3}R_{g}^{2}s^{2}\right)$$

The values of *I(0)* and *R*_*g*_ can be extracted from the y-axis intercept and the slope of the linear region of a Guinier plot (ln [*I(s)*] versus *s*^*2*^), respectively (Fig. 2C). The determination of *R*_*g*_ using this plot is not trivial as the fitting range is limited by the experimental set-up (the minimum s is defined by the size of the beam and the beamstop) and by the validity of the approximation (2) (generally, up to *s* < 1.3/*R*_*g*_). Still, reliable automated methods such as that employed by the program *AUTORG* (Petoukhov et al., 2007) have been developed to deliver high-throughput at modern synchrotron SAXS beamlines. Alternative approaches have recently been proposed [5].

Solutions of macromolecules often exhibit concentration dependent behavior. In non-ideal solutions, strong attractive or repulsive inter-particle interactions modulate the recorded scattering intensity particularly at low angles (s < 1 nm^−1^), influencing the parameters extracted from the SAXS curve (Fig. 2B). The Guinier plot provides qualitative information of the behavior of a sample and can be used to assess sample quality. Thus, samples containing non-specific aggregates yield scattering curves and Guinier plots with a sharp increase in intensity at very small *s*, while inter-particle repulsion decreases the intensity. In cases where the interactions are pronounced an additional assessment of sample monodispersity using other methods such as dynamic light scattering is highly recommended.

The contribution of the interparticle interactions to the scattering intensity can be assessed by measurements at different concentrations (typically, a stock solution at about 5–10 mg/ml and 2–3 dilutions). From such a series it is usually possible to identify the concentration where inter-particle interactions are negligible or to extrapolate the data to infinite dilution to obtain an “ideal” curve for further structural analysis.

Molecular-mass, *MM* can be estimated from the extrapolated forward scattering *I(0)* e.g. by comparison with a standard such as bovine serum albumin or glucose isomerase. This estimate requires normalization against the solute concentrations for the two measurements, and the accuracy of the *MM* estimate is limited [6]. An alternative approach involves absolute calibration using the scattering of water as a standard [7].

The hydrated particle volume (*V*_*p*_) can be obtained from the data on a relative scale. Assuming a uniform electron density inside the particle, *V*_*p*_ is estimated following Porod's equation (Porod, 1982):

(3) $$\begin{array}{l} V_{p}=2\pi^{2}I\left(0\right)/Q,\\ Q=\int_{0}^{\propto }s^{2}I\left(s\right)\cdot ds \end{array}$$

where *Q* is the so-called Porod invariant.

For real macromolecules the electron density is not uniform, however, at sufficiently high *MM* (>30 kDa), the subtraction of an appropriate constant from the scattering data generates a reasonable approximation to the scattering of the corresponding homogenous body. The particle volume, *V*_*p*_ provides an alternative estimate of the *MM* independent of uncertainties in the sample concentration. Typically, for a globular protein *V*_*p*_ (in nm^3^) is 1.5–2 times the *MM* (in kDa), and this approach is utilized in on-line processing pipelines (eg, SASFLOW) [8] and Web servers [9].

Fourier transformation of the scattering intensity yields the distance distribution function, *p(r)*:

(4) $$p\left(r\right)=\frac{r^{2}}{2\pi^{2}}\int_{0}^{\infty }s^{2}I\left(s\right)\frac{\sin\;sr}{sr}dr$$

which allows one to graphically display the peculiarities of the particle shape (Fig. 2C). For example, globular particles yield bell-shaped profiles with a maximum at approximately *D*_max_*/*2 and multi-domain particles often yield profiles with multiple shoulders and oscillations corresponding to intra and inter subunit distances. Computation of *p(r)* is not straightforward as a limited range of *I(s)* is available (from *s*_min_ to *s*_max_), and direct Fourier transformation of the scattering curve from this finite number of points is not possible. A solution to this problem is the indirect Fourier transformation method first proposed by O. Glatter [10]. In this approach *p(r)* is parameterized in the range [0, *D*_max_], with *D*_max_ being a user defined variable, and the coefficients describing *p(r)* are determined to have a smooth function providing the best fit to the experimental data. At present, automated programs are available based on an indirect transform program GNOM [11], which also find optimal *D*_max_ values (program *AUTOGNOM* [12]). Given the *p(r)* function, *I(0)* and *R*_*g*_ can be directly computed from it, and their values are typically more accurate than those obtained from a Guinier analysis.

Information about protein folding/flexibility can be obtained from the Kratky plot (*s*^*2*^*I(s) vs s*) [13], [14]. Folded globular proteins typically yield a prominent peak at low angles, whereas unfolded proteins show a continuous increase in *s*^*2*^*I(s)* with *s*. Flexible multi-domain proteins can also potentially be identified from the Kratky plot, displaying a mixture of characteristic features of both folded and unfolded proteins. Similar qualitative information can also be retrieved from a Porod-Debye plot (*s*^*4*^*I(s) vs s*^*4*^) [15]. Further rapid information about the object can be obtained by screening against a database of calculated scattering curves from all deposited structures in the PDB [16], [17], or against the recently launched SASBDB database of deposited scattering profiles and models [18]. A more direct approach is to take advantage of modern methods of *ab initio* and rigid-body reconstruction/refinement from scattering data and combine this information with other complementary structural and biochemical data.

## 3 Structure determination directly from SAXS data: *ab initio* reconstruction

While the overall parameters were once the only information derived from such experiments, advances in computational methods have now made it possible to also determine reliable low resolution (1–2 nm) three-dimensional structures. Robust *ab initio* modeling procedures have been developed to limit over-parameterisation and to take advantage of the oversampling of typical SAXS data sets [19], [20], [21], The number of independent parameters and thus degrees of freedom associated with an experimental SAXS profile can be estimated following the Shannon sampling theorem [22] and is typically of the order 10–20. An approach to estimate the number of so-called Shannon channels from experimental data has been developed and implemented in the program *SHANUM* [23], where the reliable data range for analysis is computed for a given SAXS profile. Once a meaningful data range has been identified the SAXS data can be used to drive an *ab initio* reconstruction algorithm, using a target function based on the discrepancy between the experimental and computed scattering profile is the χ^2^ criterion:

(5) χ2=1K−1∑j=1K[μI(sj)−I(sj)expσ(sj)]2

where *I*_exp_(*s*) is the experimental scattering, *K* is the number of experimental points, *σ*(*s*_*j*_) are standard deviations and *μ* is a scaling factor.

Early SAXS *ab initio* approaches used an angular envelope function to describe the shape of particles, which was further developed into a series of spherical harmonics [24], [25]. Improvements in computational power then led to a resurgence in bead modeling methods, arguably now the most popular of the *ab initio* approaches, where more detailed reconstructions are possible [21], [26], [27]. These methods, also referred to as dummy-atom modeling, reconstruct particle shape as densely packed beads inside a volume typically restrained by *D*_max_. Beads are assigned a particle or non-particle phase and the scattering intensities computed using spherical harmonics. The discrepancy (χ^2^) is evaluated in Eq. (5) between the experimental and calculated scattering intensities, however, the solution is additionally constrained by a penalty term, *P(X)*, requiring that the beads must be connected and the model compact to ensure that physically feasible low-resolution structures are generated. Multiphase bead-modeling methods have also been developed [28] and are particularly useful when contrast variation data from SANS and/or multiple SAXS curves from components of a complex (e.g. a nucleoprotein complex) are available. A limitation on the effective resolution of shape determination from bead models and envelope functions is the assumption of a uniform electron density distribution within the particle. Thus scattering curves can only be fit within a restricted range (typically up to s ∼2.5 nm^−1^, i.e. a resolution of 2.5 nm, where resolution *d* is defined as *d* = 2π/s). The *ab initio* methods are usually run multiple times with different random seeds yielding varying models, and the analysis of these models allows one to detect the most reliable features [29] and, as recently proposed, also to assess the resolution of the models [30].

An alternative approach for proteins representing the molecule as an assembly of globbed dummy residues (DR) is employed in the program GASBOR [20]. The DRs represent the average scattering of an amino-acid in water and search a random gas in place of a regular grid, and as the limitation of particle homogeneity is thus lifted the data can be fit to a much higher angles than for the bead modeling programs (up to s < 10 nm^−1^). These *ab initio* approaches have been combined with NMR chemical shift data to determine particle shape and rationalise the potential interfacial regions of complexes [31].

## 4 Calculation of SAXS intensities from models

An accurate computation of solution SAXS from intensity given a three-dimensional structure requires not only scattering from the atoms constituting the particle itself but also the contribution from the solvent. The latter contains the scattering from the excluded particle volume and, for aqueous solutions, the term emerging due to the surface hydration. A general expression for the computation of the scattering intensity from a particle in solution is:

(6) $$I\left(s\right)={\langle {\left|A_{a}\left(s\right)-\rho_{b}A_{ex}\left(s\right)+\delta \rho_{h}A_{h}\left(s\right)\right|}^{2}\rangle }_{\text{Ω}}$$

where *A*_*a*_*(s)*, *A*_*ex*_*(s)* and *A*_*h*_*(s)* are, respectively, the scattering amplitudes from the particle *in vacuo*, from the excluded volume and from the hydration layer. As shown experimentally [32], the latter is typically denser than the bulk (*ρ*_*b*_) resulting in a non-zero contrast for the surface bound solvent layer *δρ*_*h*_ = *ρ*_*h*_ – *ρ*_*b*_ [33]. This layer term is essential component of scattering intensity computations in most available programs.

A relatively straight forward approach to the calculation of atomic scattering is provided by the Debye formula (Debye, 1915), however, it scales quadratically with the number of atoms being prohibitively slow for larger macromolecules. A globbic approximation can be employed to speed up computation using the Debye formula [20], [34], [35]. Arguably, the most rapid approach is the spherical harmonics approximation used in the program CRYSOL (Svergun et al., 1995). In this approach the computation time scales linearly with the size of the model and it is highly accurate up to *s* < 5.0 nm^−1^ and in many cases up to much higher resolution (up to *s* = 10-15 nm^−1^). CRYSOL operates in both predictive and fitting modes, with default parameters used for the excluded volume and the excess scattering density of the hydration layer (*δρ*_*h*_ *∼10% of the bulk*) when computing the scattering intensity from a given model. Alternative methods for the calculations of the SAXS patterns have been developed [36], [37], [38]. For the calculations at wider angles (WAXS) methods based on molecular dynamic simulations of the solvent have been proposed [39].

The incorporation of SAXS data in NMR structure determination also requires an accurate but efficient prediction of theoretical scattering given a model. A SAXS term was added to the target function of the CNS package [40], and a globbic approximation was combined with the Debye formula [41]. This reduction decreased the computation time but introduced some systematic deviations in calculated fits. A more accurate approach also implemented in CNS uses polynomial fits to help refine the positions and orientations of rigid-bodies for multi-domain proteins [42], [43]. This approach can be tailored to the complexity of the system, for example the target function includes a term derived from *R*_*g*_ that defines a distance restraint between the centers of mass of each component, and the higher angle region of the SAXS data used to refine orientation in combination with NMR based restraints (eg. RDCs, PREs). For more complex systems additional polynomial terms can be introduced to improve convergence, provided the SAXS data range is wide (ie. *s*_max_ > 2.5 nm) and the associated signal-to-noise is high across this range. Based upon these developments a number of modern NMR structure calculation packages employ similar procedures for the incorporation of SAXS data. The most popular packages providing co-refinement methods include CNS [40] and Xplor-NIH [44], [45].

## 5 Scoring models with SAXS data

The simplest approach combining NMR and SAXS data for structural studies is to use scattering data as an *a posteriori* filter or scoring method for structures solved by NMR. An important example of model validation is the case of the Josephin domain of ataxin-3, where the fit to the SAXS data was used to discriminate between two strikingly different models [46].

Hybrid modeling using SAXS data to refine the positions and orientations of rigid bodies has become a standard approach in structural biology. Programs based on spherical harmonics [24] were developed, e.g. SASREF, BUNCH and CORAL from the ATSAS suite [12], [47], [48], facilitating rapid and straightforward refinement of rigid bodies derived from crystallography, NMR or comparative modeling against experimental scattering data.

One of the first hybrid NMR/SAXS applications was the study of a multidomain polypyrimidine tract binding protein (PTB) [49]. The structures of each RRM (RNA recognition motif) domain of PTB were individually solved by NMR. SAXS data was collected for seven constructs including full-length protein and combinations of linked and isolated domains. A multi-curve global refinement against the scattering data using the NMR structures as rigid bodies revealed the elongated structure and linker conformations of PTB. Based upon the SAXS model important predictions on how PTB interacts with mRNA were made. Another seminal example is the study of complexes formed by the iron binding protein frataxin, whose role in the formation and regulation of iron-sulfur clusters and metabolism remained unclear [50]. Exhaustive *ab initio* and hybrid SAXS modeling was conducted for binary and ternary complexes, and interfacial regions suggested by the SAXS models were validated using NMR chemical shift data and mutagenesis. This work determined the molecular details of the interaction between the scaffold protein IscU and the desulphurase IscS, and established that frataxin is the key regulator modulating the formation of this complex. In so doing the authors were able to answer a long standing question regarding the function of frataxin in the formation of iron-sulfur cluster assembly.

A recent example of hybrid SAXS/NMR analysis is given by generating starting models for RDC based refinement protocols in the study of the structure and dynamics of a trimeric G-protein α subunit (Gα). Here a decreased affinity of Gα for a nanodisc encapsulated G-protein coupled receptor (GPCR) was found to be correlated with a closed Gα conformation induced by GTP [51].

The combination of SAXS and NMR has also been successfully applied in the field of molecular docking. The programs HADDOCK [52], [53], pyDockSAXS [54], [55], and FoXSDock [56] have been developed for robust screening of ligand binding and complex formation and include scoring functions for SAXS data. It has been shown that the incorporation of SAXS data yields significant improvement in the performance of these docking algorithms [57]. An interesting approach combining the ROSETTA software package [58] with distance, orientation and chemical shift restraints has been introduced that additionally performs a grid-search using SAXS as a scoring function [59].

## 6 Co-refinement with SAXS and NMR data

The first NMR/SAXS co-refinement procedure was developed for the study of γS crystallin, a 21 kDa two-domain eye lens protein [41]. This proof of principle study was subsequently followed by work on the 146 kDa complex formed between Enzyme I and HPr [60]. These two pioneering studies demonstrate that the inclusion of both RDC and SAXS data is integral to the computation of convergent structures in cases where sparse data is available. The protocol developed was also applied to hen egg white lysozyme, demonstrating that in addition to improved convergence, the accuracy of NMR structures can be significantly improved when SAXS data is introduced into the energy function driving the structure calculations [61]. It has also been shown that the addition of wide-angle scattering data (WAXS) in co-refinement with NMR and SAXS data is highly beneficial in the structure determination of multi-helical RNAs [62].

## 7 Ensemble approaches for multi-domain and flexible systems

Flexible multidomain proteins are challenging targets in structural biology. It is becoming increasingly clear that structural disorder is an essential feature of many biological processes and it is estimated that 40% of proteins in the human genome are at least partially disordered [63]. Often the flexible regions of proteins are removed to aid crystallization and facilitate structural studies, however, important information relating to function may be lost when doing this. Intrinsically disordered proteins present an additional challenge, where high-resolution structures cannot be obtained by any method, and alternative descriptions of partially ordered or *unstructured* states are required. A successful approach introduced to provide biophysical descriptions based on structure and/or lack of structure for highly dynamic and flexible systems are ensemble based methods.

**Fig. 3:**
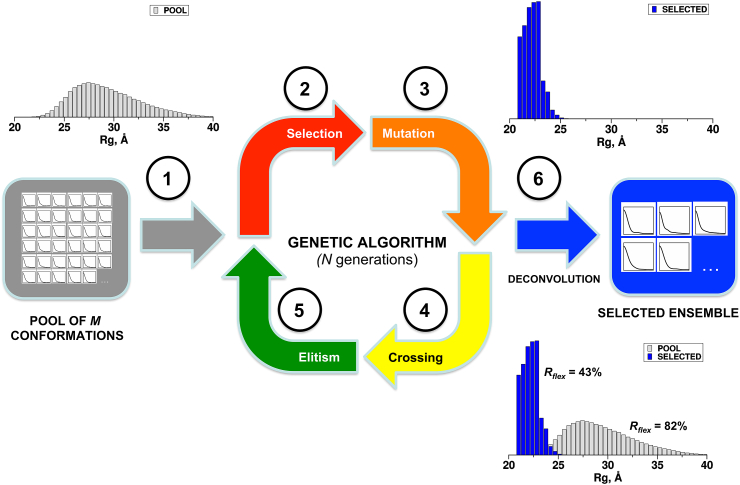
*Workflow of the Ensemble Optimization Method (EOM)*. Overall scheme of EOM. The conformational space of the system is explored through the generation of a random pool with SAXS curves computed for each conformer **(1)**. Sub-ensembles of these curves are randomly selected to form “*chromosomes”***(2)** and these chromosomes are subjected to a series of *mutation***(3)** and *crossing* steps **(4)**. The mutation stage allows swapping/replacement of curves within the current set of chromosomes and also with curves randomly selected from the pool. The crossing stage randomly selects two chromosomes from the current set and swaps a minimum of two curves to generate a new chromosome. The chromosomes that provide the best fit the experimental scattering data are evaluated at the *elitism* stage **(5)** and passed to the next generation in an iterative process. The process is repeated until the deconvolution stage **(6)**, where parameters are averaged and the final size-distributions (eg. *R*_*g*_, *D*_max_) and metrics (*R*_*flex*_, *R*_*σ*_) of selected ensembles constructed and compared with that of the initial pool.

The Ensemble Optimization Method (EOM, see Fig. 3) was the first approach to introduce the concept of ensemble fitting of experimental SAXS data for flexible systems [64], [65]. The ensemble generation procedure itself originated from the Flexible Meccano algorithm [66], developed primarily for NMR analysis of unfolded proteins.

An ensemble of conformationally polydisperse particles is approximated as a mixture according to:

(7) $$I\left(s\right)=\sum_{k}v_{k}I_{k}\left(s\right)$$

where *I*_*k*_*(s)* is the scattering intensity from the *k*-th component and *v*_*k*_ the volume fraction for that component (Konarev, Petoukhov et al., 2006). For flexible systems, given the large number of possible conformations, deconvolution of the scattering pattern into single components is unfeasible. The EOM strategy tackles this issue in an indirect way: (i) generate a large pool of possible conformations in order to approximate the accessible conformational space; (ii) compute the scattering profile for each conformation and (iii) select a subset of conformations that minimizes the discrepancy *χ*^*2*^ (Eq. (6)).

EOM uses a genetic algorithm (GA) to select subsets of conformations from the random pool that best fit the experimental data. Results are then reported as distributions of the parameters radius of gyration (*R*_*g*_), the maximum particle dimension (*D*_max_) and end-to-end distance. These distributions are then compared with those derived from the initial pool, to visually describe overall properties of biomolecules such as *compactness* and *flexibility*. Quantitative metrics describing the flexibility based on the concept of information-entropy [22] have been recently made available [65]. EOM is actively utilized to assess the flexibility of multi-domain proteins and to characterize intrinsically disordered systems [67], [68], [69], [70], [71]; see also the study of the ribosomal protein L12 in the next section.

An interesting extension of the ensemble approach combining SAXS and NMR data from pseudocontact shifts (PCS), Paramagnetic Relaxation Enhancements (PREs) and residual dipolar couplings (RDCs) is Maximum Occurrence analysis (MO) [72], [73]. In this approach the conformational heterogeneity of two-domain proteins is assessed through the estimation of a MO parameter for each conformation in an ensemble of structures. The ensembles are generated as in EOM, the SAXS profiles are computed with CRYSOL [33], and PCS, RDCs and PREs for each random conformer are calculated with CALCPARA [74]. The selection procedure seeks to not only identify conformers consistent with the SAXS and NMR data, but to additionally weight the contribution of each member of the ensemble such that each conformer cannot exceed the maximum percent of time that the system can spend in that conformation.

The MO procedure has been successfully applied to the study of matrix metalloproteinase 1 (MMP-1), an essential protein involved in collagen degradation [75]. Using paramagnetic NMR and SAXS data the authors demonstrate that the highest MO conformations of MMP-1 (MO ∼50%) are those where the catalytic and hemopexin-like domains are not in close contact. Closed conformations, as observed crystal structures, exhibit low MO (∼20%). It had been unclear just how the compact MMP-1 structures previously solved would facilitate collagen binding, mediated by the hemopexin-like domain, and subsequent collagenolysis by the catalytic domain. The high MO conformations identified by the ensemble procedure show the hemopexin-like domain to be significantly solvent exposed and poised to interact with the collagen triple-helix, and the catalytic domain in an orientation primed for collagen cleavage.

In addition to the approaches discussed above, several alternative ensemble based methods combining scattering and NMR data have been recently developed [76], [77], [78], [79]. Additional insights into the dynamic behavior of unfolded proteins are provided by single-molecule Förster resonance energy transfer (FRET), whereby the FRET-based nanosecond correlation spectroscopy probes long-range distances and chain dynamics in a time range inaccessible by NMR [79]. Interestingly, although NMR models are typically provided as ensembles of conformations, to our knowledge no successful attempts have as yet been reported using NMR conformers as input for SAXS driven flexibility analysis. The possible reason is that differences in the ensemble may reflect the ambiguity of the NMR structure calculation rather than macromolecular flexibility.

## 8 Combining SAXS and NMR for dynamics studies

NMR provides a powerful approach for the identification of residue specific flexibility and the location of order/disorder in proteins and polynucleotides [80]. Combining the ready applicability of SAXS to the study of flexible systems with the atomic-level information derived from NMR relaxation analysis can yield greater insight into the dynamics of physical processes.

A seminal example of the combination of SAXS and NMR relaxation analysis is that applied to the dimeric ribosomal protein L12 [81]. At the time of this study both NMR and crystal structures of the full length protein had been solved and NMR dynamics studies had been conducted, showing a disordered hinge region connecting the N- and C-terminal domains [82], [83], [84]. Using the high-resolution domain structures as input, a large conformational pool of dimeric L12 was generated and an ensemble of conformations selected by EOM [64], [65]. The comparably broad distributions of *R*_*g*_ for the pool and selected ensemble provided evidence of a highly flexible system, however, the skewed distribution of the selected ensemble to higher *R*_*g*_ clearly demonstrated the extended and partially structured nature of L12 linker conformations consistent with the experimental data. These ensembles were subsequently used to develop a model describing the overall rotational diffusion of the dimer and the motional coupling between domains. Reorientational Eigenmode Dynamics (iRED) analysis was employed [85] to extract eigenmodes from the selected ensemble and determine optimized correlation times in good agreement with experimental ^15^N spin relaxation data. This thorough analysis revealed a fascinating anti-correlated inter-domain structure, where the distances defined by the flexible linkers between the pairs of N- and C-terminal domains adopt an asymmetric conformation. This combined SAXS/NMR analysis allowed both local and global dynamics to be characterized, yielding insight into the function of L12 as it interacts with other ribosomal components and the translation machinery.

Another interesting example seeking to characterize system dynamics through the combination of NMR and SAXS data is that applied to the Josephin domain of ataxin-3 [86]. Following on from previous model validation using SAXS data, distinguishing between two competing Josephin domain structures [46], molecular dynamics simulations using RDC and chemical shift restraints (HN, Hα,Cα, Cβ, CO, and N) were conducted. From these simulations an ensemble of conformations was generated, and both SAXS and NOE data employed for validation. Large amplitude motions on the ns to ms time scale were detected for a helical hairpin in the free state of the protein, providing evidence for an open state facilitating ubiquitin binding. The energy landscapes derived from the best fitting ensembles of free and ubiquitin bound ataxin-3 showed an increased conformational entropy upon binding ubiquitin, consistent with previous NMR relaxation data [87].

## 9 Conclusions and outlook

As evident from this review, SAXS and NMR ideally complement each other for the study of biomacromolecular solutions. SAXS is not limited by size, yields overall shapes and is sensitive to domain/subunit positions, whereas NMR provides high resolution models and is sensitive to domain/subunit orientations. A SAXS experiment can be performed on the same sample used for NMR, facilitating quantification of concentration dependence and interparticle interference phenomena, and to investigate the effect of deuterated solvents or isotopic labeling on structure. Most importantly, both methods are readily applied under near native solution conditions, and can provide structural information on the dynamics and flexibility of the dissolved macromolecules. Presently, tremendous progress has been achieved in cryo-EM [88], [89], providing novel high resolution insights into the static structures of large complexes. We believe that the joint use of SAXS and NMR will be under high demand in the characterization of dynamic macromolecular systems where flexibility plays an essential role in their function.
